# The Story of Hospital Sunday

**Published:** 1920-07-10

**Authors:** Wm. S. Caw

**Affiliations:** Treasurer and Clerk. The Royal Infirmary, Edinburgh


The Editor's Letter Box—(*continued*).

### "The Story of Hospital Sunday."

*To the Editor of* The Hospital.

Sir,—Referring to the article under the above heading in your issue of June 26, wherein it is stated that the first "Hospital Sunday" was held in Birmingham in 1859, it may interest your readers to know that an annual collection has been regularly made in the churches and chapels of this city on behalf of the funds of its Royal Infirmary for the past one hundred years at least. On the petition of the managers presented each year to the Lord Provost, Magistrates, and Town Council, that body makes a recommendation that a certain Sunday be set apart for the purpose (that day having been the fourth Sunday in November for many years now), and which recommendation is universally adopted. Owing to the accounts not being given in detail in the old Minutes and the reports not having been printed in these far-away days, I am not in a position to state the actual year when the practice was commenced, but the return from this source formed a very considerable portion of the hospital's voluntary revenue a century ago.—I am, Yours faithfully,

Wm. S. Caw,
Treasurer and Clerk.

The Royal Infirmary, Edinburgh,
June 30, 1920.

*To the Editor of* The Hospital.

